# Prevalence and Risk Factors of Low Anterior Resection Syndrome in Epithelial Ovarian Cancer Surgery

**DOI:** 10.7759/cureus.23180

**Published:** 2022-03-15

**Authors:** Iqra Yasin, Afshan Saeed Usmani, Jibran Mohsin, Rehan Bin Asif, Nazish Kahlid, Aamir Ali Syed

**Affiliations:** 1 Gynecologic Oncology, Shaukat Khanum Memorial Cancer Hospital and Research Centre, Lahore, PAK; 2 Department of Surgery, Akhtar Saeed Medical and Dental College, Lahore, PAK

**Keywords:** ovarian cancer surgery, gynecologic oncology, epithelial ovarian cancer, low anterior resection syndrome, low anterior resection

## Abstract

Background

In this study, we aimed to determine the prevalence and risk factors of low anterior resection syndrome (LARS) in epithelial ovarian cancer (EOC) surgery.

Methodology

A descriptive cross-sectional study was conducted at the Gynecologic Oncology Section of the Department of Surgical Oncology, Shaukat Khanum Memorial Cancer Hospital & Research Centre, Lahore, Pakistan. Using non-probability consecutive sampling technique, all patients who underwent cytoreductive surgery involving low anterior resection for EOC between January 2016 and January 2021 were included. Patients were assessed for LARS symptoms using the LARS score, along with its risk factors. Descriptive statistics, that is, continuous variables were expressed as the median and interquartile range, while categorical variables were expressed as frequencies and percentages. The LARS score was categorized according to a two-tier model with “no or minor LARS” and “major LARS.” Univariate analyses were performed by the chi-square tests providing odds ratios and 95% confidence intervals to identify risk factors for major LARS.

Results

Overall, 95% of cases had LARS scores that fell in “no or minor LARS,” while only 5% of cases had “major LARS.” Univariate analyses relieved no statistically significant association between the occurrence of major LARS and any of the risk factors.

Conclusions

The prevalence of LARS was 5%, and no risk factors were associated with major LARS in our study population.

## Introduction

Ovarian cancer is the most common cause of cancer death from gynecologic tumors [1]. Malignant ovarian tumors include primary ovarian cancer and secondary metastatic ovarian cancer. The majority (95%) of all ovarian malignancies are epithelial ovarian carcinoma [2]. Other primary ovarian tumors include germ-cell tumors, sex cord-stromal tumors, and other rare tumor types. Common sources of metastases to the ovaries are the endometrium, breast, colon, stomach, and cervix.

Epithelial ovarian cancer (EOC) generally presents at an advanced stage due to ineffective population-based screening [3]. Treatment requires expert multidisciplinary care. An essential part of the treatment of EOC is cytoreductive surgery (CRS) aiming at complete tumor resection [4,5]. To fulfill the surgical goal of R1 resection, that is, no macroscopic residual disease, CRS often includes bowel resection, including low anterior resection of the rectum. It is a frequent component of CRS for advanced EOC, and additional bowel resections are sometimes required. The most important short-term complication in the postoperative period is anastomosis dehiscence [6]. Among long-term complications, low anterior resection syndrome (LARS) is a serious and common complication that affects the quality of life (QoL) of patients. LARS consists of symptoms associated with bowel dysfunction such as diarrhea, fecal incontinence (gases or stool), increased bowel movements and frequencies of defecation, fecal urgency, constipation, and incomplete emptying or accumulation of intestinal gases. There is a wide variation in appearance, duration, and severity of the abovementioned symptoms between patients [7,8].

A validated questionnaire consisting of five questions, the LARS score, is used to diagnose LARS. Based on the final score, patients are classified as having “no LARS,” “minor LARS,” or “major LARS” [9-12]. Data on LARS are nearly exclusively derived from rectal cancer studies. The prevalence of LARS in this rectal group of patients ranges from 25% to 80% [11]. Potential predisposing factors in the rectal group of patients include neoadjuvant or adjuvant radiotherapy, postoperative chemotherapy, low anastomosis, total mesorectal excision, temporary colostomy, and anastomotic complications [13-17].

Currently, no data on LARS in EOC are available except for recently published two European studies [18,19]. Thus, this study aims to determine the prevalence and identify risk factors for LARS in EOC patients undergoing upfront or interval debulking surgery, including large bowel resection with low anterior resection. This article was previously presented as a poster presentation at the 20th Shaukat Khanum Cancer Symposium 2021 on November 5, 2021.

## Materials and methods

This descriptive cross-sectional study was conducted in the Gynecologic Oncology Section of the Department of Surgical Oncology at Shaukat Khanum Memorial Cancer Hospital & Research Centre (SKMCH&RC) Lahore, Pakistan. All patients who underwent ovarian cancer surgery along with low anterior resection between January 2016 and January 2021 were included in this study. Patients who underwent Hartman or total colectomy procedures were excluded. A non-probability consecutive sampling technique was used. Approval from the Institutional Review Board (IRB) of SKMCH&RC Lahore, Pakistan was obtained. The study complied with the SKMCH&RC guidelines on research involving human subjects.

Patients were identified retrospectively from the Hospital Information System (HIS). All patients were assessed for LARS symptoms including its risk factors from the data available in HIS. LARS score is assigned using a standardized validated assessment comprising five questions concerning symptoms of a potential LARS (Table 1).

**Table 1:** Assessment form and interpretation for the LARS score. LARS: low anterior resection syndrome

LARS score		
1. Do you ever have occasions when you cannot control your flatus (wind)?		
	No, never	0
	Yes, less than once per week	4
	Yes, at least once per week	7
2. Do you ever have any accidental leakage of liquid stool?		
	No, never	0
	Yes, less than once per week	3
	Yes, at least once per week	3
3. How often do you open your bowels?		
	>7 times per day (24 hours)	4
	4–7 times per day (24 hours)	2
	1–3 times per day (24 hours)	0
	Less than once per day (24 hours)	5
4. Do you ever have to open your bowels again within one hour of the last bowel opening?		
	No, never	0
	Yes, less than once per week	9
	Yes, at least once per week	11
5. Do you ever have such a strong urge to open your bowels that you have to rush to the toilet?		
	No, never	0
	Yes, less than once per week	11
	Yes, at least once per week	16
	Total score:	
	Interpretation:	
	No LARS	0–20
	Minor LARS	21–29
	Major LARS	30–42

Descriptive statistics such as mean, median, frequencies, and percentages were used to present patients’ characteristics: prevalence (%) for categorical variables; median/range for metric variables without normal distribution; and mean (standard deviation, SD) for metric variables with normal distribution. For univariate analyses, the LARS score was categorized according to a two-tier model with “no or minor LARS” and “major LARS.” Univariate analyses were performed by chi-square tests with odds ratios (OR) and 95% confidence intervals (95% CI). Statistical analysis was done using SPSS version 20.0 (IBM Corp., Armonk, NY, USA).

## Results

This study included 21 patients who were diagnosed with ovarian cancer and underwent bowel resection along with resection of the primary tumor. Bowel resection as an inclusion criterion must involve low anterior resection with or without small or large bowel resection and anastomosis. All patients were enrolled in the Gynecological Oncology Section of the Department of Surgical Oncology at SKMCH&RC, Lahore, Pakistan. Patients’ characteristics are shown in Tables 2, 3.

**Table 2:** Mean and standard deviation of metric variables of patients’ characteristics. SD: standard deviation

Variable	Mean (±SD)
Age (years)	49 (±12.5)
Charlson Index	7 (±1.6)
Body mass index (kg/m^2^)	27 (±5.9)
Albumin serum level (g/dL)	4.2 (±0.5)

**Table 3:** Prevalence and frequency for categorical variables of patients’ characteristics. ASA: American Society of Anesthesiologists; ECOG: Eastern Cooperative Oncology Group; FIGO: Fédération Internationale de Gynécologie et d’ Obstétrique; LAR: low anterior resection; LARS: low anterior resection syndrome

Variable		n (%)
ASA	I	0 (0%)
	II	18 (86%)
	III	3 (14%)
ECOG	0	21 (100%)
	1	0 (0%)
Ascites	≤500 mL	21 (100%)
	>500 mL	0 (0%)
Tumor stage	FIGO III	19 (91%)
	FIGO IV	2 (9%)
Type of debulking surgery	Primary	10 (48%)
	Interval	11 (52%)
Type of bowel surgery	LAR only	16 (76%)
	LAR + large bowel	0 (0%)
	LAR + small bowel	5 (24%)
Number of anastomoses	1	16 (76%)
	>1	5 (24%)
Surgical complexity score	≤11	21 (100%)
	>11	0 (0%)
Residual disease	Yes	5 (24%)
	No	16 (76%)
Recurrence	Yes	10 (48%)
	No	11 (52%)
Anastomosis leakage	Yes	1 (5%)
	No	20 (95%)
LARS	No LARS	18 (86%)
	Minor LARS	2 (9%)
	Major LARS	1 (5%)

Out of a total of 21 cases, 10 underwent primary resection of ovarian cancer along with low anterior resection, and 11 cases received neoadjuvant chemotherapy followed by interval surgery. Overall, 16 (76%) cases had no residual macroscopic disease at the end of CRS. Low anterior resection alone was done in 16 (76%) cases, and additional small bowel resection on reversal of covering ileostomy was done in the remaining five (24%) cases. Most cases had single large bowel anastomosis, that is, 16 (76%) cases in comparison to four (19%) four that had two anastomoses (one of the large bowel and second of the small bowel), and one (5%) cases had three anastomoses (one of the large bowel and two of the small bowel). Out of the 21 cases, 10 (48%) patients experienced disease recurrence.

**Figure 1:**
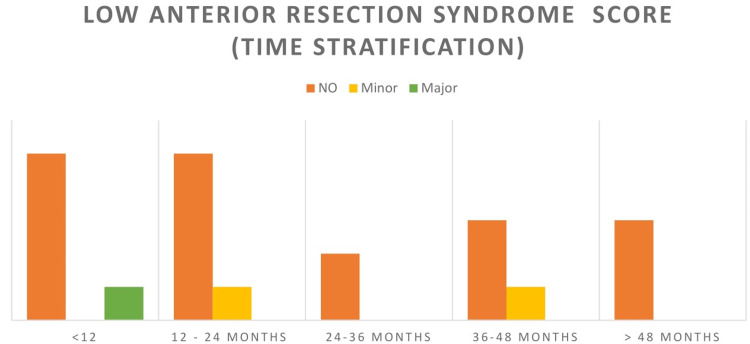
Time stratification of the LARS score. LARS: low anterior resection syndrome

Assessment for LARS symptoms revealed that 18 (86%) patients had scores that fell in “no LARS,” two (9%) in “minor LARS,” and only one case of “major LARS.” Stratification of the LARS score with respect to time elapsed since surgery is shown in Figure 1. Major LARS was seen in patients with time relapsed of less than 12 months that might change to minor or no LARS if prolonged follow-up is advised similar to other patients.

Univariate analyses, as shown in Table 4, showed no statistically significant association between the occurrence of major LARS and any of the risk factors such as age, Charlson Index, Eastern Cooperative Oncology Group (ECOG) status, body mass index (BMI), primary debulking surgery versus interval debulking surgery, stage of the tumor, number of anastomoses, surgical complexity score (SCS), presence of residual disease, anastomoses leakage, and recurrent disease. One case of major LARS was seen in a 41-year-old patient with the Charlson Index score of six, American Society of Anesthesiologists (ASA) class two and ECOG zero. Her BMI was 23 kg/m^2^, and her serum albumin level was 4.41 g/dL. The volume of ascites was less than 500 mL. Her tumor FIGO (Fédération Internationale de Gynécologie et d’ Obstétrique) stage was three, and she underwent primary debulking surgery, including low anterior resection along with small bowel anastomosis of covering ileostomy reversal for a total of two anastomoses. Her surgical complexity score was <11, and she had residual disease with no anastomotic leakage.

**Table 4:** Assessment of the risk factors for the LARS score. FIGO: Fédération Internationale de Gynécologie et d’ Obstétrique; BMI: body mass index; ECOG: Eastern Cooperative Oncology Group; LARS: low anterior resection syndrome

Variable		No or minor LARS	Major LARS	P-value
Age (years)	≤50	11	1	Not applicable
	>50	9	0	
Charlson Index	≤6	14	1	
	>6	6	0	
ECOG	0	20	1	
	≥1	0	0	
BMI (kg/m^2^)	≤25	6	1	
	>25	14	0	
Timing of surgery	Primary	9	1	
	Interval	11	0	
Tumor stage	FIGO III	18	1	
	FIGO IV	2	0	
Number of anastomoses	1	16	0	
	>1	4	1	
Surgical complexity score	≤11	20	1	
	>11	0	0	
Residual disease	Yes	4	1	
	No	16	0	
Anastomosis leakage	Yes	1	0	
	No	20	0	
Recurrent disease	Yes	9	1	
	No	11	0	

## Discussion

LARS is a common condition known to colorectal surgeons. This includes clinical manifestation in patients who undergo low anterior resection of rectal cancer. Because optimal cytoreduction in ovarian cancer patients can also include low anterior resection, LARS becomes relevant in such cases. This study aimed to explore this relevance. LARS was seen in 25-80% of rectal carcinoma cases [7]. Assuming the same prevalence in patients with ovarian cancer is not appropriate because of the differences in treatment approaches, that is, the extent of surgery [20] and non-surgical modalities of therapy.

This is the first study to assess LARS in an EOC eastern population (especially the subcontinental region of Asia) undergoing debulking surgery with low anterior resection. To date, only two studies conducted among the western population have been published on this topic [18,19]. A total of 21 patients were included in this study. All patients underwent primary tumor excision along with low anterior resection with or without small or large bowel resection.

Among baseline characteristics, the mean age of patients in this study was 10 years younger than those included in the study conducted by Kranawetter et al. [19] and 15 years younger than a comparative study conducted by Harpain et al. [18]. Mean BMI was 27 kg/m^2^ in comparison to 24 kg/m^2^ found in a multicenter comparative study [18] and by Kranawetter et al. [19]. Charlson Comorbidity Index (CCI) was higher than the rest of the two studies, that is, 7 versus 2 [18] and 4 [19], while ASA class distribution was similar, that is, ASA class 2 noted in 86% versus 75% [18] and 78% [19]. All patients in this study had ECOG zero status in comparison to 95% in the study by Kranawetter et al. [19]. While mean serum albumin level was almost similar to Kranawetter et al. [19]. Ascites of ≤500 mL was seen in all cases, while in comparison one-third of patients had >500 mL ascites in the study group of Kranawetter et al. [19].

Analyses of tumor staging showed 91% cases were of FIGO three in contrast to 55.8% in the cohort of Kranawetter et al. [19] and 80% in a multicentric comparative study [18]. The distribution of debulking surgery showed almost equal primary debulking and interval debulking. This is in contrast to 85% primary debulking reported by Kranawetter et al. [19] and 88% seen in a comparative study [18]. All surgeries were done via an open approach similar to a previously reported comparative study [18]. Low anterior resection only was done in 76% of cases, while the rest of the cases required additional small bowel resection and anastomosis. Small bowel resection and anastomosis were in addition to the reversal of covering ileostomy done in all low anterior resection cases. In the study by Kranawetter et al. [19], 73.3% of cases underwent low anterior resection only compared to 55.4% cases in a comparative study [18]. Only one anastomosis was performed in 76% of cases which was comparable to the figures reported by two previous studies [18,19]. All large bowel anastomosis were of end-to-end GI gun-guided type compared to 93% cases of similar type in the ovarian cancer cohort in a comparative study [18].

The SCS was ≤11 in all 21 cases in this study while slightly more than one-third of cases had SCS >11. Residual Disease was seen in only 20% of cases in the study of Kranawetter et al. [19] and 32% in a comparative study [18] in contrast to 24% cases of this study. Recurrence was seen in almost half of the cases in this study in comparison to around one-third of cases in both Kranawetter et al. and comparative studies [18,19]. Anastomosis leakage was seen in only one (5%) case out of the 21 cases.

The key finding of this study was the low prevalence of LARS in the subcontinental Asian population undergoing low anterior resection as part of CRS for advanced EOC. Only one case out of a total of 21 cases had major LARS, two (9%) cases had minor LARS, and the rest of 18 (86%) cases had no LARS. In comparison, around 38% of cases of Kranawetter et al. [19] and 16% of cases in the comparative study [18] had major LARS. About 18% had minor LARS in a comparative study [18] and 21% in the study reported by Kranawetter et al. [19]. No long-term side effects were observed in 66% of patients of comparative study [18] and 40.8% by Kranawetter et al. [19]. Similar results were seen in a Danish cross-sectional study [21] that showed major LARS prevalence of 18.8% in Danish 50-79-year-old females irrespective of disease, comorbidities, and medications. This study highlights the baseline risk of LARS in females of this age group even without any surgical intervention. This interpretation is significant with respect to the preoperative counseling of patients in this age group planning for upfront debulking surgery.

In addition to prevalence, the second objective of this study was to determine the risk factors for LARS in patients with ovarian cancer. Analyses of risk factors showed no risk factor because only one case had experienced major LARS (Table 4). Hence, studies with larger sample sizes are required to determine any significant association between LARS and any of its risk factors. This is in comparison to the study by Kranawetter et al. [19] that showed the presence of multiple bowel anastomosis to be an independent risk factor for major LARS that led to a four-fold increased risk for major LARS. This can be explained by the fact that multiple bowel anastomosis leading to shortened bowel might cause more liquid stool consistency, along with hyperactive postprandial response after low anterior resection. Kranawatter et al. [19] also noted that younger age was a risk factor for LARS. This finding was similar to that seen in rectal cancer cases. Age can be a risk factor due to the high prevalence of constipation in the elderly age group. In comparison, rectal cancer-associated risk factors for LARS include low anastomosis, radio or chemotherapy, different types of resections, and anastomotic leakage [13-17].

Assessing the prevalence and risk factors of LARS is important because it affects patients’ quality of life [22]. Clinicians involved in the surgical management of EOC must be aware of this phenomenon so that appropriate postoperative measures can be taken to relieve its clinical features. During follow-up visits, the main focus of gynecologic oncologists is usually to rule out recurrence [23]. However, determining the presence of LARS using a simplified LARS score can be helpful to highlight the under-reported phenomenon from both patients and doctors. It will eventually help to improve the quality of life in the long term.

Preventive and therapeutic measures for LARS are not widely available in the literature. LARS in rectal cancer can be reduced by avoidance of very low anastomosis near to anal canal, total mesorectal excision, and temporary colostomy. Data regarding preventive measures for ovarian cancer is not available yet. Therapeutic measures include medications such as loperamide hydrochloride, 5-hydroxytryptamine-3 antagonists, local supportive therapies such as transanal irrigation, pelvic floor rehabilitation, and surgical interventions such as sacral nerve stimulation. None of them are validated modalities, and response is individual [24-26].

A total of 21 patients included in this study is in comparison to 56 patients included in an ovarian cohort of a multicenter comparative cohort study and 206 patients included in the study conducted at Kliniken-Essen-Mitte, Germany, and Medical University of Vienna, Austria. The major limitation of this study was the small sample size making the assessment of risk factors and their association challenging. Hence, there is a need for further studies with a larger sample size before any definitive conclusions can be made regarding the accurate assessment of the prevalence and risk factors. Second, not all patients were assessed in a similar duration from the time of their surgery, leading to selection bias. Major LARS is a known concern in patients undergoing CRS for EOC that involves resection of the rectum. Accurate estimation of prevalence requires prospective studies with large sample sizes. The education of gynecologic oncologists is of high clinical importance.

## Conclusions

The prevalence of major LARS was 5% among patients undergoing CRS for EOC that includes low anterior resection as its component. This prevalence is lower than that seen in rectal carcinoma and EOC among western populations. Unlike multiple risk factors that are associated with LARS in rectal carcinoma, risk factors assessed in this study showed no statistically significant association between any of the described patient, tumor, or surgical parameters keeping in view the limitations of this study, especially its small sample size.
